# The effect of surgery (Ovariohysterectomy) on the plasma disposition of meloxicam following intravenous administration in dogs

**DOI:** 10.1186/s12917-016-0659-y

**Published:** 2016-02-20

**Authors:** Umit Karademir, Dilek Aksit, Cavit Kum, Hasan Erdogan, Eyup Hakan Ucar, Cevdet Peker, Cengiz Gokbulut

**Affiliations:** Department of Pharmacology and Toxicology, Faculty of Veterinary Medicine, University of Adnan Menderes, Isikli Koyu Aydin, Turkey; Department of Pharmacology and Toxicology, Faculty of Veterinary Medicine, Balikesir University, Balikesir, Turkey; Department of Internal Medicine, Faculty of Veterinary Medicine, University of Adnan Menderes, Isikli Koyu Aydin, Turkey; Department of Obstetrics and Gynecology, Faculty of Veterinary Medicine, University of Adnan Menderes, Isikli Koyu Aydin, Turkey; Department of Medical Pharmacology, Faculty of Medicine, Balikesir University, Balikesir, Turkey

**Keywords:** Dog, Meloxicam, Pharmacokinetics, Surgery

## Abstract

**Background:**

Meloxicam (MLX) is a nonsteroidal anti-inflammatory drug used in the relief of postoperative pain for human and veterinary medicine. This study was designed to investigate the effect of surgery on the plasma disposition of MLX in dogs undergoing ovariohysterectomy following a single intravenous injection at a dose of 0.2 mg/kg bodyweight. Eight crossbred bitches were used in the study. A two-phase experimental design with a 10-day washout period was used. Pre-operative MLX was administered intravenously to 8 bitches about 10 days before surgery (Phase I, control) at a dose of 0.2 mg/kg bodyweight and peri-operative MLX was administered intravenously after anaesthesia and 15 min before the start of surgery (Phase II). Blood samples were collected from all animals at various times between 1 and 96 h after the drug administrations in both phases. The drug concentrations were analysed using high performance liquid chromatography.

**Results:**

The volume of plasma MLX distribution at steady-state (Vd_ss_) of the control group (Vd_ss_: 263.0 ml/kg) was significantly greater (*P* < 0.05) compared to that of the surgery group (Vd_ss_: 149.3 ml/kg). The AUC values were higher (29.5 vs. 23.0 μg.h^2^/ml) and the CL values were lower (7.7 vs. 10.5 ml.h/kg) in the surgery group compared to the control group, respectively, but differences were not significant.

**Conclusions:**

The results of the present study indicated that surgery could alter the plasma disposition of MLX and thus the drug efficacy and side effects such as gastrointestinal ulceration, unusual bleeding and loss of kidney function/failure when repeated doses are used.

## Background

Analgesic drugs including nonsteroidal anti-inflammatory drugs (NSAIDs), opioids and local anaesthetic agents are used in dogs as peri- and post-surgery pain relievers. NSAIDs are used in veterinary medicine for treating especially musculo-skeletal and abdominal pain [7]. These drugs have antipyretic, anti-inflammatory and analgesic properties. The common mechanism of action for this class of drugs can be attributed to a blockade of the biosynthesis of prostaglandins, resulting from the inhibitionof enzyme cyclooxygenase (COX) [3, 21].

Meloxicam (MLX), belongs to the enolic acid class of NSAIDs and is also effective as antipyretic, analgesic or anti-inflammatory agent [20]. It preferentially inhibits COX-2, which is induced by inflammatory stimuli in pathophysiological conditions [9]. MLX is about 12 times more potent at inhibiting COX-2 than COX-1 activity [17].

Surgery is a duration when there are some major stresses associated with pain, cardiovascular and possibly major haemodynamic changes peri- and post-operation, especially if there is a significant blood loss [1]. In general, the pharmacokinetics of drugs in most species have been characterized in healthy animals. However, the plasma disposition of the active compound and its metabolites may be markedly altered in diseased animals or during peri- or post-operative period.

The pharmacokinetics data are available for some NSAIDs in dogs undergoing surgery and can be compared with other published data [6, 7, 11, 27]. However, to the best of our knowledge, this is the first study in which the pharmacokinetic disposition of an NSAID in dogs has been evaluated in the same animal with and without surgery. Our hypothesis was that surgery alters the plasma disposition of certain drugs including analgesics and anaesthetics administered pre- or peri-operatively. Therefore, this study was designed to investigate the effects of surgery on the plasma disposition of MLX in dogs undergoing ovariohysterectomy following a single intravenous injection at 0.2 mg/kg bodyweight.

## Results

Clinically no adverse effects were observed during and after the MLX administration in dogs. The pre-operative biochemical and haematological parameters were within the normal ranges. Mean age, bodyweight, duration of surgery and the selected surgical variables during anaesthesia are shown in Table 1. The heart rate, respiratory rate and end tidal CO_2_ values were 122.17 ± 10.6 beats/min, 8.5 ± 0.9 breaths/min and 42.06 ± 7.3 mmHg during anaesthesia, respectively. The albumin levels of each dog in the control group were similar to its own post-surgery plasma albumin concentration. In addition, the mean albumin concentration in the control group (2.43 ± 0.32 g/dl) was not significantly different (P: 0.719) compared with that observed in the surgery group (2.50 ± 0.23 g/dl).

**Table 1:** Mean (±SD) values of general characteristics and selected surgical variables during anaesthesia of eight dogs undergoing ovariohysterectomy

Characteristic	Mean ± SD
Operation time (min)	29 ± 7.9
Age (year)	1.4 ± 0.4
Bodyweight (kg)	18.25 ± 4.4
Time from MLX injection to start of surgery (min)	15 ± 0.0
Heart rate (per min)	122.17 ± 10.6
Respiration rate (per min)	8.5 ± 0.9
End tidal CO2 (mmHg)	42.06 ± 7.3
Oxyhaemoglobin (%)	>98

The analysis yielded linear regression lines ranging from 0.025 to 10 μg/ml of MLX with a correlation coefficient of 0.999. The limits of detection and quantification of the MLX assay were 0.005 μg/ml and 0.02 μg/ml, respectively. The mean recovery was 98.32 % (inter assay CV = 2.09 %) and the accuracy ranged from 96 % to 103 %.

**Fig 1:**
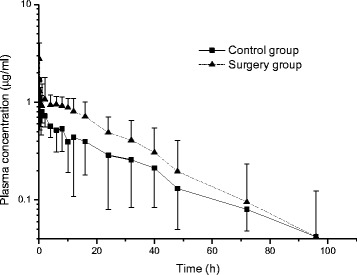
Semi log plot of mean (±SD) plasma concentrations *vs.* time curves of meloxicam in control and surgery groups following intravenous (0.2 mg/kg) administration to bitches (*n* = 8)

**Fig 2:**
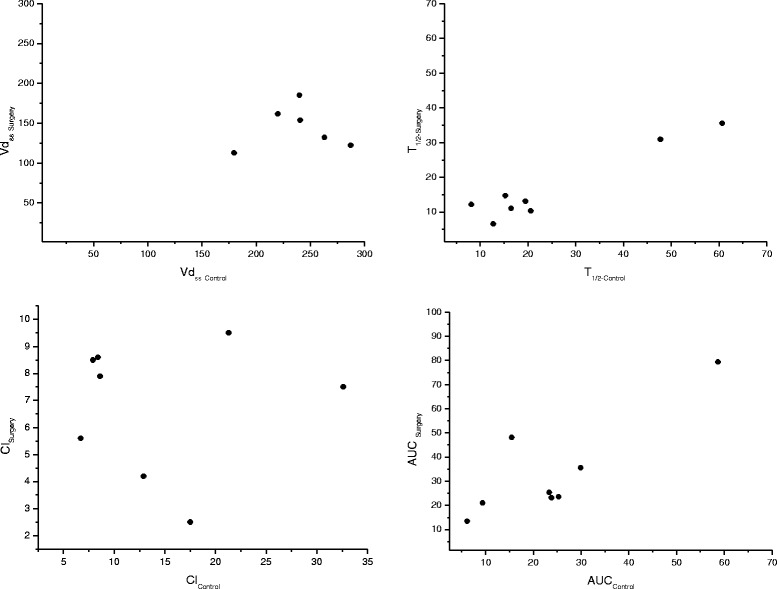
The scatter plots of some pharmacokinetic parameters (Vd_ss_, T_1/2_, Cl and AUC) in control group *vs*. surgery group following intravenous (0.2 mg/kg) administration of meloxicam to dogs (*n* = 8)

Pharmacokinetic parameters of MLX following intravenous administration (0.2 mg/kg) are given in Table 2. Semi logarithmic plot of the mean plasma concentration *vs*. time curves are shown in Fig 1. In additions, the scatter plots of the pharmacokinetic parameters (Vd_ss_, T_1/2_, Cl and AUC) in control *vs.* surgery group are presented in Fig. 2.

**Table 2:** Median pharmacokinetic parameters with lower and upper confidence intervals of meloxicam in control and surgery groups following intravenous (0.2 mg/kg) administration to bitches (*n* = 8)

Parameters	Control group	Surgery group	*P* values
T_1/2λz_ (h)	17.21 (12.8–40.1)	12.77 (9.8–24.3)	0.289
C_0_ (*μ*g/m*l*)	1.05 (0.7–9.92)	5.3 (1.9–14.2)	0.298
AUC_last_ (*μ*g.h/m*l*)	21.2 (10.8–30.8)	28.5 (18.7–45.6)	0.176
AUC_0→∞_ (*μ*g.h/m*l*)	23.0 (12.4–41.0)	29.5 (19.4–51.5)	0.322
AUMC_0→∞_ (*μ*g.h^2^/m*l*)	698 (378–2815)	588 (329–2150)	0.959
Cl_*B*_ (m*l*.h/kg)	10.5 (6.0–20.6)	7.7 (4.9–11.35)	0.191
Vd_ss_ (m*l*/kg)	263.0 (211–470)	149.3 (122.4–172.7)	0.016*
MRT_0→∞_ (h)	28.1 (19.5–56.0)	20.7 (14.4–34.3)	0.303

C_0_: plasma concentration at time 0, AUC_0→∞_: area under the (zero moment) curve from time 0 to infinity, T_1/2λz_: terminal half-life, AUMC_0→∞_: area under the moment curve from time 0 to infinity; Cl_*B*_: total body clearance of drug; Vd_ss_: volume of distribution at steady-state; MRT_0→∞_: mean residence time

*The kinetic parameters in control group are significantly different (*P* < 0.05) from the surgery group

The differences in most pharmacokinetic parameters between the control and surgery groups were not significant, however, variability was high for each phase. Nevertheless, the terminal half-life (T_1/2λz_) and total body clearance (Cl_*B*_) values were longer and higher, but the area under the curve (AUC) value was smaller in the control group (T_1/2λz_: 17.21 h, Cl_B_: 10.50 ml.h/kg and AUC: 23.00 μg.h/ml) compared to the surgery group (T_1/2λz_: 12.77 h, Cl_B_: 7.70 ml.h/kg and AUC: 29.50 μg.h/ml), respectively. The volume of MLX distribution at steady-state (Vd_ss_) of the control group (Vd_ss_: 263.00 ml/kg) was significantly larger (*P* < 0.05) compared to that of the surgery group (Vd_ss_: 149.30 ml/kg). The Vd_ss_ value of each individual animal decreased in phase I compared with that of the same animals in phase II. Moreover, T_1/2λz_, AUC and MRT values in 7 out of 8 dogs were altered in the post-surgery group compared to the control group.

## Discussion

The plasma concentration of MLX in dogs following intravenous administration exhibits slight fluctuations, especially in the early hours of the administration in both phases of the present study. Similar observations were also reported by studies performed recently in dogs, cats and horses subjected to ketorolac treatment [7, 11, 27]. The reason of such characteristics in plasma concentrations is not clear and probably not associated with the anaesthetic agents and surgical procedures employed in the experiments, since similar fluctuations were observed in the animals of the control group.

There is a reasonable body of literature evaluating peri- or post-operative analgesic effects and plasma dispositions of some NSAIDs in dogs undergoing surgery [4, 7, 8, 12, 24]. However, to our knowledge this is the first study in which the pharmacokinetics of an NSAID in dogs have been evaluated in the same animal with and without surgery. Although, all animals under surgery receive at least 1 drug during the operation for pre-anesthetic medication, anesthesia or analgesia in the surgery unit, there is no information available on the alterations in drug disposition following surgery in veterinary medicine. During the surgery, many hemodynamic and metabolic alterations such as fluctuating hemodynamics, alteration of protein catabolism and anabolism may occur. The changes in circulation, blood volume, extracellular fluid and blood protein levels, such as α1-acid glycoprotein and albumin may affect the distribution of drugs used during the peri- and post-operative periods [18].

The kinetic parameters of MLX observed from the control group in the present study exhibits a low apparent volume of distribution (263.00 ml/kg) and a long plasma half-life (17.21 h) and residence time (28.10 h) in dogs following the intravenous administration. These findings are in agreement with the results of a previous study [5], regarding the values of V_ss_ (320 ml/kg), T_1/2_ (24.0 h), MRT (34.8 h) and AUC (21.5 μg.h/kg) in healthy dogs.

The differences in most pharmacokinetic parameters between the control and surgery groups were not significant, however, variability was high for each phase. The dogs in the control group were demonstrated to have a prolonged half-life (17.21 h *vs* 12.77 h), smaller AUC (23.00 μg.h/ml *vs* 29.50 μg.h/ml) and greater body clearance (Cl_B_) (10.50 ml.h/kg *vs* 7.70 ml.h/kg) when compared to themselves in the surgery group. Moreover, the volume of MLX distribution at steady-state in the control group (Vd_ss_: 263.00 ml/kg) was significantly greater than that of the surgery group (Vd_ss_: 149.30 ml/kg). Volume of distribution (Vd_ss_) is one of the most important parameters for the evaluation of the pharmacokinetic characteristics of the drugs and depends on many factors, including the physicochemical properties of the drug, binding strength of plasma proteins, binding capacity to the other tissues. The reason for low levels of Vd_ss_ measured in surgery group is probably associated with major haemodynamic changes during the surgery and/or drug-drug interaction. The hepatic activity increases during the post-operative period. Serum albumin turnover elevates, drug-albumin binding characteristics alter and the level of serum proteins, including albumin decreases dramatically for a period following surgery [18]. In addition, it has been reported that the serum albumin level was significantly reduced in dogs with inflammation compared with the healthy dogs [2]. Besides, high plasma albumin binding of MLX could be altered by the anaesthetic drugs, since it has been reported that the plasma proteins (albumin, globulin) decreases in under isoflurane anaesthesia in flying fox [15]. Both the duration and the intensity of drug action with the free drug concentration being the active compound are affected by the level of protein binding [26]. In these circumstances, the protein binding is influenced, particularly for the drugs which have more than 90 % protein bound [18]. The protein binding ratio of MLX in dogs was reported as 97 % [5] and MLX exhibits a high degree of binding to albumin [16]. Generally, the reduction of plasma protein leads the increase of distribution volume and clearance of drugs via the decrease in the plasma protein bindings. However, the mean albumin concentration in the control group (2.46 g/dl) was not significantly different (P: 0.719) compared with that observed in the surgery group (2.48 g/dl) in the present study. Consequently, the difference on Vd_ss_ of MLX is probably not related to the different plasma albumin level between the control and surgery groups. The reason for lower Vd_ss_ in the surgery group was however unclear. It is probably related to the other factors such as drug-drug interaction which may have changed the drug-protein binding capacity in the surgery group since atropine sulphate, propofol and isoflurane were combinely used for premedication and as anaesthesia. Also ovariohysterectomy may have affected reproductive hormones and thus the disposition of the drugs. In addition, the reason for lower Vd_ss_ in the surgery group could be, at least in part, due to smaller Cl_B_ and MRT as Vd_ss_ = Cl_B_ × MRT. Moreover, there was a limitation since the control and surgery phases (I and II) were arranged in a sequence, but not in a crossover fashion. This experimental design may potentially induce a period effect. However, this is judged to be unlikely as a 10-day washout period was considered to be sufficiently long [22].

In the present study, the effect of surgical operation on the plasma disposition of MLX was investigated in only crossbred and female dogs. However, the plasma disposition of MLX may be different in pure-bred and between male and female dogs. The elimination half-life of naproxen in beagles (T_1/2_: 35 h) was much shorter [25], compared with that of observed in mongrels (T_1/2_: 74 h) [13]. This difference probably reflects a breed difference, since it is known that beagles metabolise drugs more rapidly than other breeds [14]. Besides, it has been reported that, female dogs showed lower AUC and elimination T_1/2_ values of MLX, compared with male dogs [5].

## Conclusions

Vd_ss_ of MLX in the control group is significantly greater compared to that of the surgery group. Generally limited or lowered distribution may decrease the drug exposure into tissues. However, the consequent reduction in volume of distribution leads to higher plasma concentrations of MLX. Besides, higher plasma concentration of the increased unbound MLX fraction in plasma may increase risk of side effects such as gastrointestinal ulceration, unusual bleeding and loss of kidney function/failure when repeated doses are used. The change in the disposition of some drugs pre-, peri- and post-operatively administered for pre-medication, anaesthesia or prevention of post-operative infections may result in changing efficacy and/or increasing side effects or toxicity.

## Methods

### Experimental animals

A total of 8 client-owned, cross-bred bitches, 2–4 years old and weighing 18.25 ± 4.4 kg were used in the study. Animals were judged to be healthy on the basis of physical examinations and haematological and biochemical blood tests. All animals were housed in single boxes and each dog was identified by natural markings. Water was supplied *ad libitum* and animals were fed a standard commercial diet twice daily with an appropriate quantity of feed during the experimental period. The animals were acclimated for 5 days before the study and food was withdrawn 12 h before the drug administrations in pre- and peri- surgery.

The study was designed as a two-phase trial with a 10-day washout period as this time duration was reported to be adequate for ensuring the clearance of the drug in dogs [22]. A bolus of MLX was administered intravenously to each dog about 10 days before the surgery (Phase I, control) at a dose of 0.2 mg/kg bodyweight and peri-operative MLX was administered intravenously after the anaesthesia and 15 min before the start of the surgery (ovariohysterectomy) (Phase II) at the same dose 0.2 mg/kg bodyweight. MLX administration and blood sampling were performed through the different cephalic veins of each dog. All animals were enrolled after written consent of the owners. The experimenters were blinded to the pharmacological treatment, while processing data and making exclusion decisions. All procedures were carried out under a protocol approved by the Animal Ethics Committee of Adnan Menderes University (Reference number: 64583101/2014/170). All sections of this paper adheres to the ARRIVE Guidelines for reporting animal research [19].

### Pre-surgical and surgical procedures

All dogs in phase II were administered to the following anaesthetic protocol: premedication by subcutaneous atropine sulphate (0.045 mg/kg bwt, Teknovet Atropine; Teknovet, Turkey), induction by intravenous bolus of propofol (4 mg/kg bwt, Propofol 1 %; Fresenius, Fresenius Kabi) administered for inducing the anaesthesia. After intubation, the anaesthesia was maintained with isoflurane (Isoflurane USP; Adeka, Turkey). Different levels of isoflurane were used during anaesthesia, to maintain an appropriate depth of anaesthesia based on clinical assessment; heart rate (HR), invasive blood pressure (IBP), muscle relaxation, the degree of nystagmus and response to the surgery were monitored.

MLX (Bavet Meloxicam; Bavet, Istanbul-Turkey) was administered intravenously as a bolus at a dose of 0.2 mg/kg bodyweight after intubation and 15 min before the start of the surgery.

After the dog was anesthetized, a standard aseptic preparation was performed, which was followed by elective ovariohysterectomy via a routine ventral midline approach. Same surgeon performed all ovariohysterectomy operations. Respiration rate, HR, IBP, EtCO_2_, oxyhaemoglobin saturation, electrocardiography (lead II), and body temperature were recorded every 5 min during the operation period using an Utech monitor (VS2000V Vital Signs Monitor; Utech Co. Ltd., China).

### Sample collection

Heparinized blood samples (3 ml) were collected by cephalic venepuncture using a 20 G catheter (B&D Medical Systems; Istanbul, Turkey) prior to drug administration (0 h), 5, 15, 30, 45 min and 1, 2, 4, 6, 8, 10, 12, 16, 24, 32, 40, 48, 72 and 96 h after the administration of MLX. Blood samples were centrifuged at 3000 g for 20 min and plasma was transferred to plastic tubes (5 ml). Heparinized drug-free blood samples for analytical method development and validation process were collected from other dogs not included in the study. All the plasma samples were stored at −20 °C until the estimations of drug concentrations.

### Analytical procedures

The parent compounds of MLX in dog plasma were analysed using validated high performance liquid chromatography (HPLC) following a liquid-liquid phase extraction procedure. Plasma concentrations of MLX were measured by minor modifications of the methods described by Eniko et al. [10].

Stock solution (100 μg/ml) of pure standard of MLX (Dr. Reddy’s Laboratory, India) was first prepared using acetonitrile: water (20:80) as solvent. The resulting stock was then further diluted to produce 0.05, 0.5, 1, 2.5, 5 and 10 μg/ml solutions for calibration as standard curves and to add to the drug-free plasma samples to determine the recovery.

The mobile phase consisted of acetonitrile : 1 % aqueous glacial acetic acid (40:60, v/v) and was delivered (Agilent 1260 Series binary pump; Waldron, Germany) at a flow rate of 1.2 ml/min. An analytical column (Zorbax CN, 5 μm, 4.6 mm x 250 mm; Agilent Technologies, Wilmington, DE) was used for the analysis of MLX. The eluate was continuously monitored using a photodiode array detector (Agilent 1260 Series Technologies; Waldron, Germany) at a wavelength of 360 nm.

The method for MLX analysis in plasma was validated before the start of the analysis of the study samples. The chromatographic peak of MLX was identified with the retention times of its analytical standard. Recovery of the molecule was determined by comparison of the peak areas from the spiked plasma samples with the areas resulting from direct injections of the analytical reference standard. The inter- and intra-assay precisions of the extraction and chromatography procedures were evaluated by processing replicate aliquots of drug-free dog plasma samples containing known amounts of the drug on different days. Calibration graphs were prepared (linear range 0.025-10 μg/ml). The slope of the lines between peak areas and drug concentration was determined by least squares linear regression and showed a correlation coefficient between 0.9987 and 0.9999. The detection limit of the molecule was established with HPLC analysis of blank plasma spiked with the analytical standard and measuring the baseline noise at the peak retention time. The limit of detection (LOD) and limit of quantification (LOQ) were determined for the HPLC method. The limits were determined based on the standard deviation amongst response and slope of the curve at the lowest concentrations.

Plasma albumin concentrations of all samples of each animal were determined by autoanalyser (Sinnowa D280, China), using bromocresol green (Archem A2011, İstanbul, Turkey) as described by Lolekha and Charoenpol [23].

### Pharmacokinetics and statistical analysis of data

The plasma concentration *vs*. time curve obtained after the treatment was fitted with the WinNonlin software program (version 5.2, Pharsight Corp., Mountain View, California, US). The pharmacokinetics parameters for each animal were estimated using non-compartmental model analysis. The trapezoidal rule was used to calculate the area under the plasma concentration time curve (AUC).

The pharmacokinetic parameters are reported as median with lower and upper confidence intervals. The characteristics of dogs (Table 1), the plasma albumin concentrations (Table 2) and the plasma concentrations *vs*. time curves of MLX (Fig. 1) were expressed as mean ± SD. The pharmacokinetic parameters and plasma albumin concentrations were statistically compared with a Wilcoxon’s signed rank test a nonparametric approach for paired data. All statistical analyses were performed by using MINITAB for Windows (release 12.1, Minitab Inc., State College, PA, USA). Mean values were considered significantly different at *P* < 0.05.

## Abbreviations

AUC_0→∞_: area under the (zero moment) curve from time 0 to infinity
AUMC_0→∞_: area under the moment curve from time 0 to infinity
C_0_: plasma concentration at time 0
Cl_*B*_: total body clearance of drug
COX: cyclooxygenase
MLX: meloxicam
MRT_0→∞_: mean residence time
NSAIDs: nonsteroidal anti-inflammatory drugs
T_1/2λz_: terminal half-life
Vd_ss_: volume of distribution at steady-state
